# Surgery for pulmonary lesions in patients with a history of urinary tract transitional cell carcinoma

**DOI:** 10.1186/s13019-024-02607-z

**Published:** 2024-03-12

**Authors:** Ryu Kanzaki, Akihiro Nagoya, Seiji Taniguchi, Hiroto Ishida, Kenji Kimura, Eriko Fukui, Toru Kimura, Takashi Kanou, Naoko Ose, Soichiro Funaki, Masato Minami, Eiichi Morii, Yasushi Shintani

**Affiliations:** 1 Department of General Thoracic Surgery, Osaka University Graduate School of Medicine, L5-2-2 Yamadaoka, Suita-City, Osaka 565-0871 Japan; 2 Department of Pathology, Osaka University Graduate School of Medicine, Suita, Japan

**Keywords:** Lung cancer, Surgery, Pulmonary metastasis, Urinary tract transitional cell carcinoma

## Abstract

**Background:**

There has been little information on the actual diagnosis of pulmonary lesions in patients with a history of urinary tract transitional cell carcinoma (TCC) and short- and long- outcomes of pulmonary resection for these patients.

**Methods:**

In the present study, the data of 37 consecutive patients with a history of TCC who underwent pulmonary resection for solitary pulmonary lesions were reviewed, and the clinical factors and short- and long-term outcomes were analyzed.

**Results:**

The study population included 35 male patients, and 2 female patients. The mean age was 72.5 years. Twenty patients (80%) were smokers and showed a high incidence of chronic obstructive pulmonary disease. Pulmonary lesions and primary TCC were detected simultaneously in 5 patients and metachronously in 32 patients. The median interval between treatment for primary TCC and the detection of pulmonary lesion was 43 months. The mean tumor diameter was 23 mm. The types of resection included lobectomy (n = 19), segmentectomy (n = 8), and partial resection (n = 10). Twelve of 37 patients (32%) developed postoperative complications. The pathological diagnoses included primary lung cancer (n = 28), pulmonary metastasis from TCC (n = 7), and others (n = 2). The 5-year overall survival rate for all patients was 72%. The 5-year overall survival rate of patients with primary lung cancer was 74%, while that of patients with pulmonary metastasis from TCC was 57%.

**Conclusions:**

Surgery can be proactively considered for treating pulmonary lesions in patients with a previous history of TCC, as it provides favorable long-term outcomes.

## Background

Worldwide, urinary tract transitional cell carcinoma (TCC) is the 6th most common cancer in males [1]. A certain proportion of patients with TCC who underwent surgery experience pulmonary metastasis [2, 3]. Because smoking is a risk factor for both TCC and primary lung cancer [4, 5], co-occurrence of primary lung cancer as a second cancer in patients with TCC is not rare (2.6–3.1%) [6, 7].

The differential diagnosis of solitary pulmonary lesions in patients who experienced TCC includes primary lung cancer, pulmonary metastasis from TCC, other neoplasms, and inflammation. Thus far, there have been no reports on the actual diagnosis of pulmonary lesions in patients with a history of TCC. In the present study, we report the final diagnosis of solitary pulmonary lesions that occurred in patients with a history of TCC, and analyzed short- and long-term outcomes of pulmonary resection in these patients.

## Patients and methods

### Ethical statement

The study protocol was approved by the Ethics Review Board for Clinical Studies at Osaka University (control number 18237, 28th June 2022 and 18373, 27th May 2022). Requirement for written informed consent was waived by the Ethics Review Board.

### Patients

The present study was a retrospective analysis of 37 consecutive patients with a history of TCC who underwent pulmonary resection for solitary pulmonary lesions in our hospital between 2003 and 2020. Information on comorbidities were collected from medical charts. The diagnostic criteria for COPD were established in the “guidelines for the diagnosis and treatment of COPD 3rd edition” by the Japanese Respiratory Society [8].

### Treatment strategy and the evaluation of surgical outcomes

When pulmonary metastasis from TCC was confirmed or suspected preoperatively, resection was performed in cases that fulfilled the following criteria: (1) the pulmonary lesion was deemed to be completely resectable; (2) the absence of apparent mediastinal lymph node metastasis was determined by a preoperative radiological examination; (3) the metastatic disease was limited to the lungs (4) locoregional control of the primary TCC was achieved; and (5) the patient was in good overall general condition and had an adequate respiratory function to tolerate lung resection. The type of resection was selected according to the size and location of the tumor and the overall general conditions and respiratory function of the patient. When metastasis from TCC was confirmed or suspected preoperatively, lesser resection was preferably selected as long as curative resection was possible. When primary lung cancer was confirmed or suspected preoperatively, lobectomy and mediastinal lymph node dissection was generally performed. Sublobar resection and/or omission of mediastinal lymph node dissection were performed in patients with an impaired general condition or respiratory function. Sublobar resection with curative intent is normally indicated for patients with ground glass opacity (GGO) lesions or solid dominant lesions of < 1.5 cm in diameter. Our treatment algorithm for limited resection for small-sized lung cancer was described previously [9].

Follow-up after pulmonary resection was generally based on the findings from chest computed tomography (CT), a physical examination, and laboratory blood tests performed every 6 to 12 months after lung resection. Follow-up information was obtained from hospital medical records. The time interval between pulmonary resection and the latest follow-up examination in the present study ranged from 6 to 153 months (median: 62 months).

All of the specimens obtained from pulmonary resection were reviewed by pathologists. When it is difficult to make a diagnosis according to the morphology determined by HE staining, immunohistochemical staining of TTF-1, Napsin-A, CK7, CK20, and GATA3 are added. TTF-1+ and/or Napsin-A+ and/or CK7+/CK20-strongly suggest lung cancer, while GATA3+ and/ or CK7+/CK20+ strongly suggest TCC [10, 11].

### Statistical analyses

The statistical analyses were performed using the JMP Pro 14 software program (SAS Institute, Berkley, CA, USA). Data are expressed as the mean ± standard deviation. Survival rates were analyzed by the Kaplan–Meier method using the date of pulmonary resection as the starting point. *P* values of < 0.05 were considered to be statistically significant.

## Results

### Patient characteristics and preoperative diagnosis

The patient characteristics are summarized in Table 1.

**Table 1:** Patient characteristics

Characteristics	No. of patients
Sex	
Male	35 (95%)
Female	2 (5%)
Age (years)	
Mean ± SD	72.5 ± 7.1
Range	58–89
Smoking status	
Smoker	31 (84%)
Never smoker	6 (16%)
Brickman Index	
Median	900
Range	0–2500
Medical history	
Malignancy besides urinary tract TCC	12 (32%)
COPD	22 (59%)
Hypertension	15 (41%)
Coronary artery disease	6 (16%)
Diabetes mellitus	5 (14%)
Cerebral infarction	2 (5%)
Interstitial lung disease	2 (5%)
CKD	12 (32%)
Atherosclerotic diseases	8 (22%)
Atrial fibrillation	5 (14%)

CKD, chronic kidney disease; COPD, chronic obstructive pulmonary disease; SD, standard deviation; TCC, transitional cell carcinoma

All patients had been treated for primary TCC with curative intent, as summarized in Table 2. The median interval between treatment for primary TCC and the detection of pulmonary lesion was 43 months (range 0–346 months).

**Table 2:** Information on primary urinary tract transitional cell carcinoma

Characteristics	No. of patients
Primary site	
Urinary bladder	28 (76%)
Renal pelvis	2 (5%)
Multiple tumor in both bladder and renal pelvis or ureter	7 (19%)
Pathologic stage of primary TCC*	
0a,0is,I	10 (40%)
II	2 (8%)
III	4 (16%)
IV	1 (4%)
Unknown	8 (32%)
Histologic grading of primary TCC	
G1	3 (8%)
G2	11 (30%)
G3	11 (30%)
Unknown	12 (32%)
Treatment for TCC	
TUR-Bt	23 (62%)
Cystectomy	7 (19%)
Nephroureterectomy	6 (16%)
Cystectomy and nephroureterectomy	1 (3%)
Interval between treatment for TCC and detection of pulmonary lesion (months)	
0 (synchronous)	5 (14%)
< 24	10 (27%)
24–60	10 (27%)
> 60	12 (32%)
Urinary bladder relapse of TCC before lung resection	
Yes	8 (22%)
No	29 (78%)

TCC, transitional cell carcinoma; TUR-Bt, trans-urethral resection of bladder tumor

**Fig. 1:**
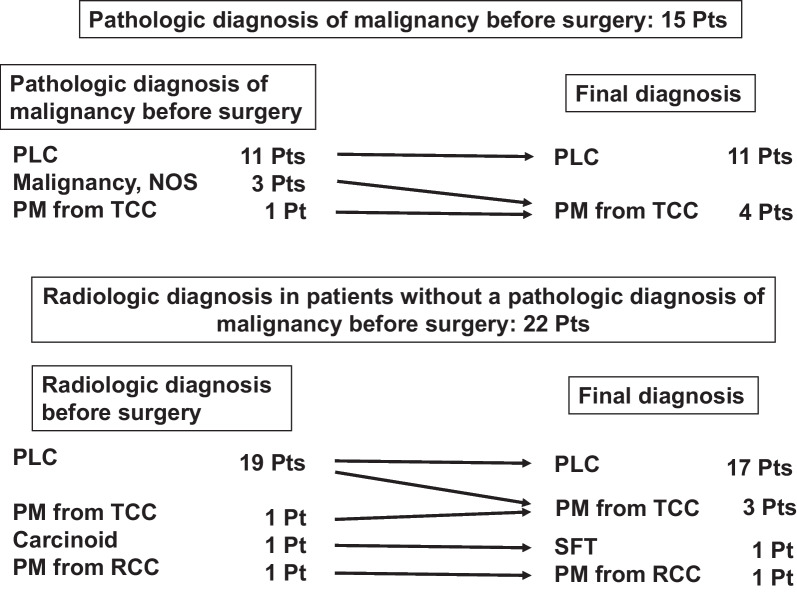
The relationship between the preoperative diagnosis and postoperative final diagnosis. NOS, not otherwise specified; PLC, primary lung cancer; PM, pulmonary metastasis; RCC, renal cell carcinoma; SFT, solitary fibrous tumor; TCC, transitional cell carcinoma

A pathologic diagnosis of malignancy of pulmonary lesions was confirmed before surgery in 15 patients (transbronchial lung biopsy, n = 11; computed tomography [CT]-guided percutaneous core needle biopsy, n = 2 endobronchial ultrasound-guided transbronchial needle aspiration, n = 1). Eleven patients were diagnosed with non-small-cell lung cancer, three were diagnosed with malignancy not otherwise specified, and one was diagnosed with pulmonary metastasis from TCC. The radiologic diagnoses in patients without pathologic confirmation of malignancy before surgery were primary lung cancer in 19 patients, pulmonary metastasis from TCC in 1, carcinoid in 1, and pulmonary metastasis from renal cell carcinoma (RCC) in 1 patient (Fig. 1).

### Operative factors and short-term outcomes

Operation-related factors are summarized in Table 3. Complete resection was achieved in all patients. There was no perioperative mortality. Blood transfusion was performed in one patient.

**Table 3:** Operation-related factors

Characteristics	No. of patients
Preoperative chemotherapy for lung resection	
Yes	2 (5%)
No	35 (95%)
Presence of GGO component by preoperative CT	
Yes	12 (32%)
No	25 (68%)
Location of tumor	
Right upper lobe	9 (24%)
Right middle lobe	1 (3%)
Right lower lobe	10 (27%)
Left upper lobe	16 (43%)
Left lower lobe	1 (3%)
Tumor size (mm)	
Mean ± SD	23 ± 11
Range	6–56
Approach	
Thoracotomy	17 (46%)
VATS	20 (54%)
Type of resection	
Lobectomy	19 (51%)
Segmentectomy	8 (22%)
Partial resection	10 (27%)
Lymph node dissection	
Not done	14 (38%)
Sampling	6 (16%)
Hilar	3 (8%)
Mediastinal	14 (38%)
Operation time (min)	210 ± 99
Blood loss (g)	208 ± 382

CT, computed tomography; GGO, ground glass opacity; SD, standard deviation; VATS, video-assisted thoracoscopic surgery

Twelve of 37 patients (32%) developed postoperative complications (prolonged air leak [PAL], n = 5; atrial fibrillation, n = 3; and postoperative bleeding, acute exacerbation of interstitial pneumonia, ileus, myocardial infarction, surgical site infection, and respiratory dysfunction, n = 1 each [some patients suffered from multiple complications]).

### Final diagnosis and CT findings

Immunohistochemistry was performed to make a final diagnosis in 9 of 37 patients. The pathological diagnoses included primary lung cancer, n = 28 (adenocarcinoma, n = 18; squamous cell carcinoma, n = 7; pleomorphic carcinoma, n = 2; and small cell carcinoma, n = 1), pulmonary metastasis from TCC, n = 7; solitary fibrous tumor, n = 1 and pulmonary metastasis from RCC, n = 1. The pathological stages of lung cancer were classified as follows: IA, n = 17; IB, n = 8; IIA, n = 1 and IIB, n = 2. The relationship between the preoperative diagnosis and postoperative final diagnosis were shown in Fig. 1. Three patients diagnosed with malignancy not otherwise specified preoperatively were finally diagnosed with pulmonary metastasis from TCC. Two of the 19 patients with radiologic diagnoses of primary lung cancer without a pathologic diagnosis of malignancy were finally diagnosed as pulmonary metastasis from TCC.

**Fig. 2:**
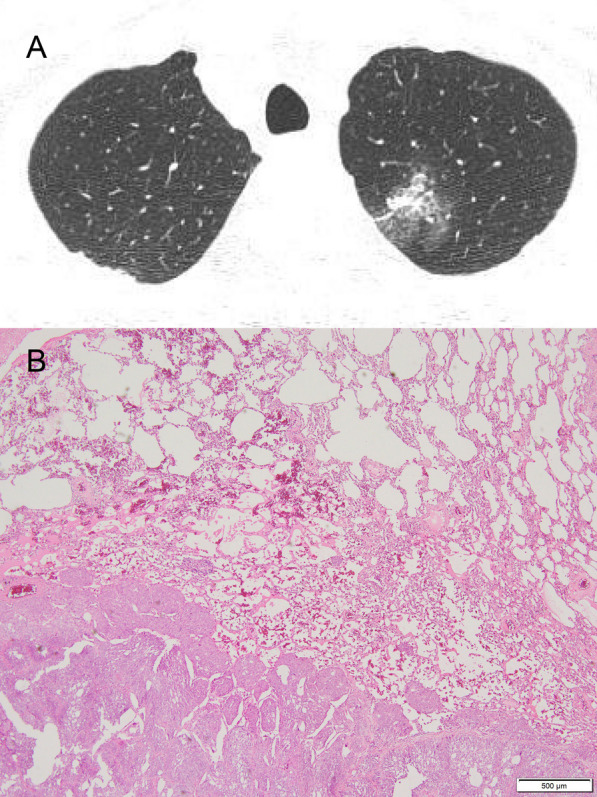
A representative case of a patient with pulmonary metastasis from transitional cell carcinoma with a ground-glass opacity component on preoperative computed tomography. **A** A pulmonary nodule containing both solid and ground-glass opacity components was located in the left upper lobe on preoperative computed tomography. **B** HE staining of the pulmonary metastasis from transitional cells, low-power view. Inflammatory cell infiltration into the alveolar spaces is observed adjacent to the site of pulmonary metastasis

According to the preoperative CT findings, 12 pulmonary lesions with a GGO component were postoperatively diagnosed as lung cancer in 10 patients and as pulmonary metastasis from TCC in 2 patients, while 26 pulmonary lesions without a GGO component were postoperatively diagnosed as lung cancer in 18 patients, pulmonary metastasis from TCC in 5 patients, SFT in 1 patient, and pulmonary metastasis from RCC in 1 patient. In both cases with pulmonary metastasis from TCC that had a GGO component, the presence of the GGO component was due to inflammation of adjacent tissue from pulmonary metastasis (Fig. 2).

### Long-term outcomes

**Fig. 3:**
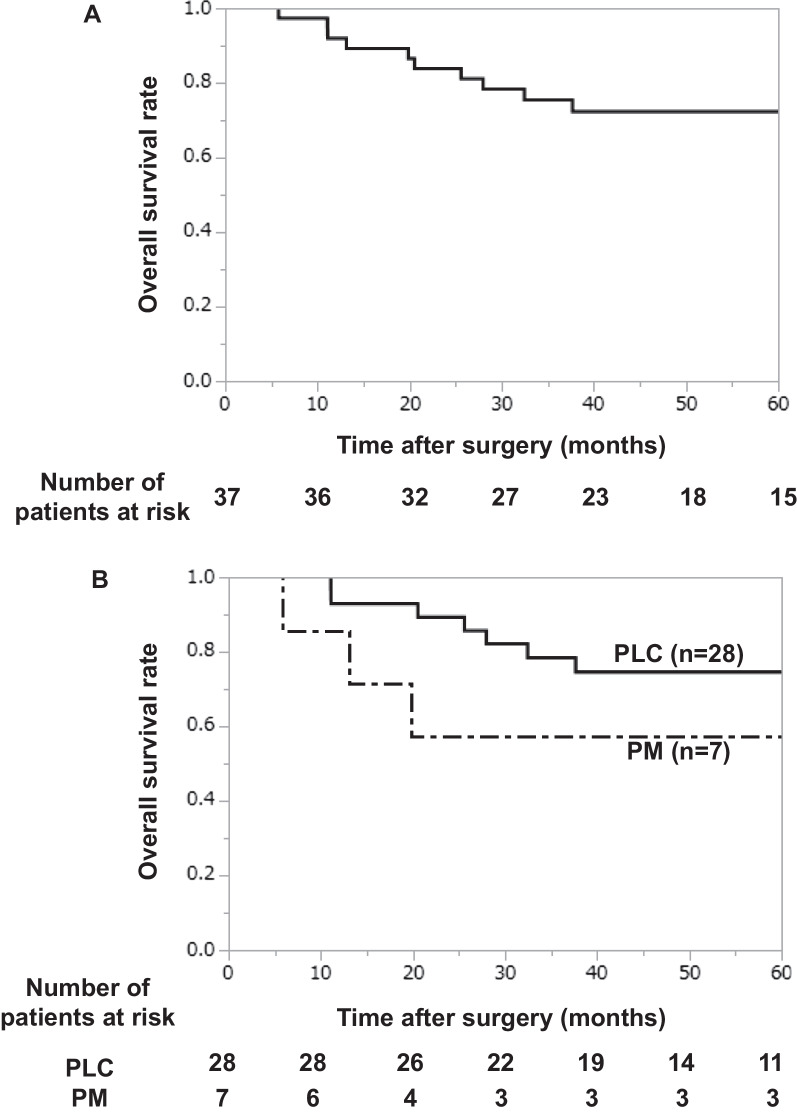
Long-term outcomes of pulmonary resection for pulmonary lesions in patients with a history of urinary tract transitional cell carcinoma. **A** Overall survival after pulmonary resection in all patients. The 5-year overall survival rate was 72%. **B** The survival rate of patients after the resection of primary lung cancer and pulmonary metastasis from urinary tract transitional cell carcinoma. The 5-year survival rate of patients with primary lung cancer was 74%, and that of the patients with pulmonary metastasis from urinary tract transitional cell carcinoma was 57%. PLC, primary lung cancer; PM, pulmonary metastasis

At the time of writing this report, 27 patients are alive and 10 patients have died. The causes of death were as follows: TCC, n = 4; lung cancer, n = 3; and other disease, n = 3. The 5-year overall survival (OS) rate of the whole study population was 72%. The 5-year rate of patients with primary lung cancer was 74%, while that of patients with pulmonary metastasis from TCC was 57% (Fig. 3).

## Discussion

In the present study, nearly four-fifths of the pulmonary lesions in patients with a history of TCC who underwent pulmonary resection were primary lung cancer. These patients had a high incidence of comorbidities, and a relatively high rate of postoperative complications. Pulmonary resection provided favorable long-term results for these patients.

In the present study, patients with a history of TCC who underwent pulmonary resection had a relatively higher incidence of comorbidities such as COPD, chronic kidney disease (CKD) and other cancer. These poor general conditions are considered to be associated with smoking. The postoperative complication rate in the present study (32%) was relatively high. In particular, 5 patients (14%) suffered from PAL. This observation was in agreement with previous studies that demonstrated a higher incidence of postoperative pulmonary complications in patients with COPD who underwent lung cancer surgery [12, 13]. When operating on these patients, surgical procedures that prevent PAL should be applied [14].

The differential diagnosis of pulmonary lesions based on radiological imaging is often difficult. In the present study, 2 out of 19 patents (11%) whose disease was diagnosed as primary lung cancer according to a radiological diagnosis without a pathologic diagnosis of malignancy were finally diagnosed with pulmonary metastasis from TCC postoperatively, as shown in Fig. 1. A radiological diagnosis of solitary pulmonary lesions is made based on various factors, including presence of a GGO component which is defined as a radiological finding in CT that consists of a hazy opacity that does not obscure the underlying bronchial structures or pulmonary vessels [15]. It is reported that metastatic pulmonary cancer can present as nodules including a GGO component [16, 17].

The theoretical mechanisms underlying the presentation of pulmonary metastasis as mixed GGO are as follows: (i) lepidic growth of metastatic cancer cells (i.e., metastatic cancer cells replacing the normal alveolar epithelium resembling lepidic-pattern lung cancer); (ii) metastatic cancer cells expand into the interstitium of the lung (i.e., lymphovascular space of the lung); (iii) bleeding spreads into the adjacent lung; and (iv) inflammation of the adjacent lung. The pathological findings of lepidic growth of metastatic cancer cells have long been observed. Several authors reported the radiologic features of GGO and corresponding pathological findings. GGO and the corresponding pathological findings of lepidic growth of metastatic cancer cells were reported in melanoma and pancreatic cancer [18, 19]. In the present study, 2 of 7 patients (29%) with pulmonary metastasis from TCC had GGO component on preoperative CT. The presence of the GGO component was due to inflammation of the adjacent tissue from pulmonary metastasis in both of the cases. Based on these findings, pulmonary metastasis can present as mixed GGO on CT; thus, the diagnosis of pulmonary metastasis should not be excluded when mixed GGO is observed in patients with a history of cancer.

Lung lesions detected in patients with a history of TCC should be diagnosed as precisely as possible before treatment in order to make an appropriate intraoperative decision. As shown in Fig. 1, 31 of 37 patients were correctly diagnosed before surgery in the present study. An intraoperative diagnosis is useful for compensating for the preoperative diagnosis. The overall diagnostic accuracy of the intraoperative frozen section diagnosis for unconfirmed pulmonary nodules with previous malignancy was reported to be 83.3% [20]. In the present study, the 5-year OS rate for all patients was 74%. We believe that surgery should be the first choice of treatment for solitary pulmonary lesions in patients with a history of TCC who can tolerate surgery based on the favorable long-term outcomes in the present study.

This study was associated with several limitations. First, the study analyzed patients who were treated over a decade, during which time the radiological and therapeutic modalities changed. In particular, the outcome of surgery for pulmonary metastasis was largely affected by the assessment of extrapulmonary metastasis. Second, this study was performed using a database of patients who underwent pulmonary resection, which did not include patients with pulmonary lesions with a history of TCC who did not undergo pulmonary resection. Therefore, the present study cannot attest to the incidence of lung cancer among TCC survivors. Thirdly, this is a retrospective study and is associated with the inherent limitations of such studies. Fourth, the number of patients was small. In the future, a multicenter prospective study should be conducted to determine the ratio of primary lung cancer to pulmonary metastasis from TCC in patients with SPN and a history of TCC.

## Conclusion

Nearly three-fourths of pulmonary lesions in patients with a history of TCC who underwent pulmonary resection were primary lung cancer. These patients had a high rate of comorbidities, and a relatively high rate of postoperative complications, especially prolonged air leak. Surgery can be proactively considered for treating pulmonary lesions in patients with a previous history of TCC, as it provides favorable long-term outcomes.

## Data Availability

All the data used in the present study are preserved in Department of General Thoracic Surgery, Osaka University Graduate School of Medicine and are available from the corresponding author on reasonable request.

## Abbreviations

CKD: Chronic kidney disease
CT: Computed tomography
COPD: Chronic obstructive pulmonary disease
GGO: Ground glass opacity
NOS: Not otherwise specified
OS: Overall survival
PAL: Prolonged air leak
PLC: Primary lung cancer
PM: Pulmonary metastasis
RCC: Renal cell carcinoma
SBRT: Stereotactic body radiotherapy
SD: Standard deviation
SFT: Solitary fibrous tumor
TCC: Transitional cell carcinoma
VATS: Video-assisted thoracic surgery
